# Implementation of prehabilitation in colorectal cancer surgery: qualitative research on how to strengthen facilitators and overcome barriers

**DOI:** 10.1007/s00520-022-07144-w

**Published:** 2022-05-25

**Authors:** Thea C. Heil, Elisabeth J. M. Driessen, Tanja E. Argillander, René J. F. Melis, Huub A. A. M. Maas, Marcel G. M. Olde Rikkert, Johannes H. W. de Wilt, Barbara C. van Munster, Marieke Perry

**Affiliations:** 1 Department of Geriatric Medicine, Radboud Institute for Health Sciences, Radboud University Medical Center, Nijmegen, The Netherlands; 2 Department of Geriatric Medicine, Gelre Hospitals, Apeldoorn, The Netherlands; 3 Department of Geriatric Medicine, Elisabeth-Tweesteden Hospital, Tilburg, The Netherlands; 4 Department of Surgery, Radboud Institute for Health Sciences, Radboud University Medical Center, Nijmegen, The Netherlands; 5 Department of Internal Medicine, University Medical Center Groningen, University of Groningen, Groningen, The Netherlands

**Keywords:** Colorectal cancer, Prehabilitation, Qualitative research, Implementation,, Complex intervention

## Abstract

**Purpose:**

Prehabilitation is increasingly offered to patients with colorectal cancer (CRC) undergoing surgery as it could prevent complications and facilitate recovery. However, implementation of such a complex multidisciplinary intervention is challenging. This study aims to explore perspectives of professionals involved in prehabilitation to gain understanding of barriers or facilitators to its implementation and to identify strategies to successful operationalization of prehabilitation.

**Methods:**

In this qualitative study, semi-structured interviews were performed with healthcare professionals involved in prehabilitation for patients with CRC. Prehabilitation was defined as a preoperative program with the aim of improving physical fitness and nutritional status. Parallel with data collection, open coding was applied to the transcribed interviews. The Ottawa Model of Research Use (OMRU) framework, a comprehensive interdisciplinary model guide to promote implementation of research findings into healthcare practice, was used to categorize obtained codes and structure the barriers and facilitators into relevant themes for change.

**Results:**

Thirteen interviews were conducted. Important barriers were the conflicting scientific evidence on (cost-)effectiveness of prehabilitation, the current inability to offer a personalized prehabilitation program, the complex logistic organization of the program, and the unawareness of (the importance of) a prehabilitation program among healthcare professionals and patients. Relevant facilitators were availability of program coordinators, availability of physician leadership, and involving skeptical colleagues in the implementation process from the start.

**Conclusions:**

Important barriers to prehabilitation implementation are mainly related to the intervention being complex, relatively unknown and only evaluated in a research setting. Therefore, physicians’ leadership is needed to transform care towards more integration of personalized prehabilitation programs.

**Implications for cancer survivors:**

By strengthening prehabilitation programs and evidence of their efficacy using these recommendations, it should be possible to enhance both the pre- and postoperative quality of life for colorectal cancer patients during survivorship.

## Introduction

Perioperative decline in functional capacity and condition in older patients with colorectal cancer (CRC) is not only caused by surgery itself, but also by the passive “waiting list period” before surgery [1]. The incidence and severity of decline in functional capacity could be reduced by prehabilitation [2, 3]. This is the process including assessments and interventions to establish a baseline functional level, identify impairments, and increase functional capacity between the time of cancer diagnosis and surgery [4]. Prehabilitation programs can be unimodal, focusing on optimizing physical condition solely, or multimodal, focusing on optimizing physical condition, nutritional status, and reduction of stress and anxiety [5]. Other components, such as smoking cessation, preoperative treatment of anemia, or medication reconciliation are also integrated as part of these programs. It is expected that a multimodal approach has synergistic effects resulting in better overall outcomes compared to unimodal approaches [6].

Prehabilitation in high-risk patients undergoing colorectal cancer surgery has shown promising results such as shorter hospital stay, reduction in postoperative complications, less functional decline, and improvements in health-related quality of life [7–10]. Therefore, prehabilitation for patients with colorectal cancer is being increasingly applied in hospitals.

However, prehabilitation is a complex intervention (as it comprises multiple components acting interdependently with evidence from heterogenous patient populations) and evaluation of such a complex intervention is difficult due to challenges in developing, identifying, documenting, and reproducing the intervention [11]. The complexity of prehabilitation and its evaluation are illustrated in the diversity in prehabilitation program designs (generally pragmatically and in line with what is achievable at the local setting) and differences in patient selection between the clinical trials. This leads to contradictory evidence regarding (cost-)effectiveness of prehabilitation [12–16] as well as to lack of generalizability of the results [17].

Because most clinical trials fail to evaluate their development and process phase, and almost no studies focus on implementation and effectiveness in daily practice, it is difficult to create a better understanding of the process of implementation of prehabilitation and the opportunities to improve. Qualitative research concerning the perspectives of professionals involved in prehabilitation in research settings as well as in daily care can help to understand how prehabilitation is delivered and which elements are perceived as important or problematic [17]. Previous qualitative studies already highlighted four key barriers for healthcare professionals to implementing a prehabilitation program: knowledge, resource, inconsistent practice, and poor patient engagement. However, there is a lack of documented facilitators [18]. Therefore, the aim of this interview study was to identify expected and perceived barriers and facilitators, in order to provide clinicians who want to implement prehabilitation in colorectal cancer surgery in their local setting, with practical recommendations*.*

## Methods

### Design

A qualitative study using semi-structured interviews with healthcare professionals involved in preoperative colorectal cancer surgery was performed.

### Participants

Colorectal cancer surgeons, specialized (oncological) nurses, physical therapists, and dieticians were approached by email to participate in the interviews. At least two participants from each profession were purposefully selected. Eligible participants, both with and without prehabilitation experience, were identified based on a previous study of our group [19]. Prehabilitation was defined as a preoperative program with at least the aim of improving physical fitness and nutritional status.

Background information on the interviewees was collected regarding medical specialty, age, gender, years of working experience, and yes or no experiences with prehabilitation in colorectal cancer care. The total number of interviews needed was guided by thematic saturation. Thematic saturation was defined as the point where no new relevant knowledge from the data analysis was obtained. In practice, this was defined as the point where no new codes were assigned during open coding. The saturation was determined independently of the represented professions, which means that irrespective of the professional asked, no new codes were added [20].

### Research team

The multidisciplinary research team consisted of nine researchers, most of whom were also clinicians. Two geriatricians (HM, MO), one internist-geriatrician (BM), one colorectal cancer surgeon (HW), one general practitioner with extensive qualitative research experience (MP). Two epidemiologists (ED, RM), one with research experience in cancer (p)rehabilitation (ED) and the other one in resilience management (RM). Two PhD students, conducting research in the field of prehabilitation and colorectal cancer, who are also medical doctor (TA, TH).

### Data collection

The interviews were conducted between September 2019 and October 2020 at a place suitable for participants, or by telephone during the COVID-19 outbreak. Interviews lasted between 20 and 30 min. The interviews were independently conducted by one of three researchers (ED, TA, TH). A critical appraisal of previous literature on prehabilitation and implementation research was conducted to gain a comprehensive and adequate understanding of the subject [21]. These knowledge was used to compile the preliminary topic list in a meeting among members of the research group (Supplement 1). To confirm the coverage and relevance of the content, the topic list was adopted during the study whenever this was required based on preliminary data analysis.

All interviews were audiotaped and transcribed verbatim.

### Data processing and analysis

Anonymized transcripts were imported into ATLAS.ti 8.4.20. After eight of in total thirteen interviews, scripts were independently coded by the first and second author using an open coding procedure [22].Codes were then compared and discussed until consensus was reached into a preliminary code book including potential barriers and facilitators. Each consecutive interview was coded directly afterwards, again independently by both the first and second author. After comparison, discussion, and consensus, the code book was adapted if needed. Disagreements were resolved through discussion and in consensus with the last author if necessary.

Thereafter, in a research team meeting, codes were compared to existing literature on models for dissemination and implementation research to determine the relationships between codes and to provide practical recommendations that are in line with clinical practice. The Ottawa Model of Research Use (OMRU) was selected to categorize the obtained codes for the purpose of practice recommendations [23]. The OMRU model was selected because it is a process model, specifically an action model, providing practical guidance in the planning and execution of implementation endeavors [24]. Using the OMRU model, key concepts as initial coding (sub)categories were identified. Codes that could not be categorized based on the model were organized in a new (sub)category [23].

#### The Ottawa Model of Research Use

The OMRU framework is an action-based model for studying implementation of healthcare innovations [24, 25]. The framework proposes to study six key components: innovation, environment, adopters, strategies for transferring evidence into practice, the use of evidence, and health-related and other outcomes of the process. These components are connected to each other through the process of evaluation [23]. The framework guides the assessment of potential barriers and facilitators to prehabilitation with regard to the innovation (prehabilitation), environment (hospital), adopters (health care professionals and patients), and if possible, also the strategies the interviewees identified for the implementation of prehabilitation. By incorporating specific barriers and facilitators into tailored strategies, the identified barriers can be overcome and facilitators enhanced. Also, suggestions are provided to monitor and evaluate the impact of implementation [23, 24].

## Results

Thirteen interviews were conducted and included five surgeons (S1-5), three specialized nurses (SN1-3), three physical therapists (PT1-3), and two dieticians (D1-2) (Table 1). Interviewees worked in different hospitals, one academic hospital and four non-academic hospitals. Three interviewees had no experience with prehabilitation. The other interviewees had experience with prehabilitation, mainly in research setting, from less than 1 year to a maximum of 3 years.

**Table 1:** Baseline characteristics interviewees

Profession	Age	Sex (M/W)	Years of professional experience	Experiences with prehabilitation in colorectal cancer care (yes/no)
Surgeon (*n* = 5)	41–58	3/2	4–23	3/2
Specialized nurse (*n* = 3)	49–59	0/3	6–12	3/0
Dietician (*n* = 2)	53–59	0/2	25–35	2/0
Physical therapist (*n* = 3)	36–58	2/1	11–23	2/1

*M*, men; *W*, women

Data are presented as number or range

Tables 2 and 3 contain an overview of all coded barriers and facilitators, respectively. Also illustrative quotes with accompanying professional background of the interviewee are shown. We found no clusters of codes related to the professional background of the interviewees observed.

**Table 2:** Identified barriers for prehabilitation application in clinical practice by professional discipline, including illustrative quotes

Identified barriers	Surgeon (*n* = 5)	Specialized Nurse (*n* = 3)	Physical Therapist (*n* = 3)	Dietician (*n* = 2)	Illustrative quotes
Combining appointments is difficult due to different work activities	**x**				*S: “Logistically it is quite complex. For example: patients have to come back to the physical therapist on Monday. But we cannot see the patient on Monday because we are scheduled to be on the operation room for the whole day.”*
Contradictory and low quality of scientific evidence for (cost-) effectiveness	**x**		**x**		*S: “I’m not convinced yet. While the need of prehabilitation is not currently proven, the implementation takes a lot of effort. Perhaps better selection of patients who really need it should be prioritized”*
Costs must be financed immediately while yields are not (directly) clear	**x**				*S: “The benefits are hard to observe. Readmissions often occur for different medical specialties. On the other hand, costs of a prehabilitation program are clear: the salary of a physical therapist and dietician must be paid.”*
Counseling patients is time consuming			**x**	**x**	*PT: “You have to plan a lot of appointments, that is satisfying, but also costs a lot of energy.”*
Differences in patients’ resilience and training opportunities	**x**	**x**	**x**		*S: “… For example for a 85 years old frail patient with a rollator, frequently cycling is not going to work… However I am convinced that even repetitive sit-to-stand exercises could work.”*
Effectiveness difficult to prove due to heterogeneity of patient population	**x**				*S: “… It is difficult to determine one outcome measure for the entire group because the main goal is to get the patient back to the level before operation.”*
Goal and content of prehabilitation program is unclear			**x**	**x**	*D: “Things did not always go well at the beginning. Patients did not understand what they opted into and ultimately they did not want to come over anymore for something like prehabilitation.”*
Healthcare professionals are unaware of (importance of) prehabilitation program	**x**		**x**		*PT: “We had a patient who went to another medical specialty, not involved in prehabilitation. That person thought it was quite a tough program and said to the patient: ‘Take it easy’ and the patient followed this advice.”*
Healthcare system is not adapted, including availability of paramedics in hospital	**x**	**x**			*S: “There is a trend to downsize paramedical care within the hospital and to relocate it outside the hospital…. As a result, new physical therapists are no longer allowed to be hired in the hospital where I work.”*
Indirect costs for patients (e.g. travel expenses)		**x**			*SN:“Patients have to come to the hospital three times a week and you have to pay quite a lot of parking costs here. Of course, if you can’t afford that, that could be a barrier.”*
Lack of program organization evaluation		**x**		**x**	*D:“There is no structured meeting to discuss logistic bottlenecks.”*
Multidisciplinary consultation is time consuming	**x**		**x**	**x**	*S: “It would be yet another multidisciplinary consultation… You also need all kinds of (para)medics for that.”*
Operating room planning takes precedence over prehabilitation program	**x**	**x**	**x**	**x**	*PT: “Recently, we had a lot of people who were scheduled for prehabilitation, came to our intake and just heard that the surgery is planned next week.”*
Patients are unable to visit hospital frequently		**x**	**x**		*PT: “… Sometimes patients experience practical barriers to take part in a prehabilitation program because they have walking difficulties and no transportation to the hospital.”* *SN: “It would be nice if patients could train close to home as well. Sometimes people start with prehabilitation but are not able to finish because they have to travel three times a week to the hospital.”*
Quality of care for colorectal surgery is already high with low complication rates	**x**				*S: “In the hospital where I work, the frail older patients with colon carcinoma are not doing so badly postoperative.”*
Uncertainty which group benefits (most) from prehabilitation	**x**		**x**		*PT: “I think it must be for a certain group of patients, patients who really need it. Because otherwise you might train patients who are already physically strong enough. They do improve in condition, but they have less tendency to end up in the ‘critical zone’ postoperatively.”* *PT: “I think everyone benefits from prehabilitation and I would like to offer it to everyone … or at least some form of it.”*
The idea that sedentary behavior is necessary when cancer is diagnosed		**x**	**x**		*SN: “In particular, the older patients have been raised with the idea that when you are sick you have to rest in order to get well.”*
The idea that tumor should be removed as soon as possible		**x**	**x**		*SN: “…Sometimes patients find it difficult as they feel that surgery is being delayed when participating in a prehabilitation program. Patients prefer to have the cancer removed yesterday instead of in three weeks.”*
Time between operation indication and surgery is too short	**x**	**x**	**x**	**x**	*D: “Right now, a lot of patients drop out because the surgery is planned within 5 days after the consultation with the specialized nurse, where prehabilitation is discussed for the first time. The prehabilitation period is then too short.”*

*S*, surgeon; *SN*, specialized nurse; *D*, dietician; *PT*, physical therapist

**Table 3:** Identified facilitators for prehabilitation application in clinical practice by professional discipline, including illustrative quotes

Identified facilitators	Surgeon (*n* = 5)	Specialized Nurse (*n* = 3)	Physical Therapist (*n* = 3)	Dietician (*n* = 2)	Illustrative quotes
Accessible contact between involved healthcare professionals		x	x	x	*D: “Like the surgeons, we visit the patients when they are training with the physical therapist, allowing accessible contact.”*
Adjust patient selection during implementation based on (local) results	x		x		*S: We definitely need to evaluate if we want to select patients to whom prehabilitation is beneficial. Therefore, you need to evaluate who did improve their physical condition and who did not. We continuously aim to gain knowledge about this and apply it in practice.”*
An ambassador should persuade, enthuse, and unite coworkers	x		x		*S: “…There must be a medical specialist who says we are going to do this and organize it well. The rest will follow naturally…. If no one puts energy and time into it… nothing will happen, as the rest is merely waiting.”*
Application of prehabilitation fits in hospital strategy	x				*S: “The overarching strategy of the hospital where I work is to make the region the healthiest region in the Netherlands and everything is aimed at that. Prehabilitation fits well in this strategy and that helps a lot to get things done.”*
Awareness regarding impact of surgery on physical condition	x	x	x		*S: “….To realize that it is a major operation, that you have to be fit for…. Some patients need a wake-up call*
Both objective as well as patient reported outcomes are important for program evaluation	**x**		**x**		*“Ultimately, prehabilitation should translate into both gaining quality of life, as well as reduction of complications, mortality and costs.”*
Combining appointments on a single day	**x**		**x**	**x**	*D: “It is an intensive program for patients. Anyway, they come back to the hospital three times a week for training. The appointments of the dieticians and physical therapist are therefore already linked as much as possible.”*
Coordination of program and program appointments by a specialized nurse	**x**	**x**	**x**	**x**	*S: “We have the advantage of having a specialized nurse who follows the patient throughout the process, during all appointments with different specialists… This helps to embed these kind of processes thoroughly…”*
Delay surgery if necessary	**x**				*S: “At the moment of diagnosis you have to start treatment within a certain time period. But I think that if you decide within a multidisciplinary team that a patient benefits more by first improving their condition, I think it is justified to postpone the operation for three months. For the cancer itself, that usually doesn’t matter anymore. Unless someone has a lot of blood loss or obstruction complaints. Then you make different choices.”*
Evaluation of individual patients only in case of signaled problems or deviation from program	**x**		**x**	**x**	*PT: “… Patient progress during the program is reported in the electronic patient record and we have close contact if patients deteriorate conditionally… You can raise the alarm immediately. This is a big advantage of the close collaboration we have now*
Evidence regarding effectiveness of prehabilitation is important for program sustainability	**x**				*S: “… High-quality evaluation of the yields. Then you can take next steps. In the past, attempts were made to set up something like prehabilitation in the Netherlands, but it failed because everyone was doing something slightly different and there was no unambiguous evaluation.”*
Group activities to exchange experiences and motivate peers	**x**		**x**	**x**	*PT: “In my opinion, group activities are of exceptional added value. The interaction between patients going through the same situation is important. Everyone has the same fears and insecurities. We also hear this from patients, that they really like this interaction.”*
Guarantee financial support	**x**	**x**	**x**		*SN: “It would be nice if financial support is guaranteed, especially because both the dieticians and physical therapist need extra full-time equivalents.”*
Incorporate social environment to facilitate patient with prehabilitation program		**x**	**x**	**x**	*PT: “Social environment plays an important role. We have had patients who wanted to do something extra at home in addition to the training program. And then the family said ‘take it easy, you already trained yesterday’.”*
Implementation of digital tools for interaction and reduction of travel distance	**x**		**x**		*S: “If a patient has mobility problems, it is impossible to drive thirty kilometers. That won’t work. Maybe digital tools can be useful.”*
Include skeptical healthcare professionals in prehabilitation team from the adoption phase	**x**				*S: “There are critics among healthcare professionals, you have to ‘bring them in’ from the start and throughout the whole implementation process. In our hospital, the oncologist was critical as always. However, in the end he was the voice-over of the prehabilitation video.”*
Individualized program	**x**	**x**	**x**	**x**	*S: “It has to be tailor-made. That is very important. Even for the very frail older patient who needs a walker, even he/she can do simple chair-rise exercises.”*
Insight in movement pattern	**x**		**x**		*S: “I have had plenty of patients who thought they were fit, but actually had a quite unhealthy lifestyle. After they started cycling and eating healthy before surgery, they said: ‘I’m sleeping better and feel much fitter after surgery’…”*
Introduce prehabilitation as part of regular care	**x**	**x**	**x**		*PT: “Patients think it is just a choice to take part in a prehabilitation program, that it is not mandatory. Especially frail patients will not choose to participate if they have to come three times a week.”*
Introduce prehabilitation early in trajectory	**x**	**x**			*S: “We have the advantage that we have a specialized nurse who follows the patient from the colonoscopy at the gastroenterologist until after the surgery. As a result, prehabilitation is introduced early in the trajectory and the importance is emphasized time and over again by a familiar face to the patient.”*
Offering an intervention program close to home	**x**	**x**	**x**	**x**	*PT: “So far, most patients prefer to train near their home because it is practical. We refer patients to a physical therapist nearby and provided the training program…”*
Patients are able to improve their self-reliance instead of just waiting	**x**	**x**	**x**	**x**	*SN1: “… What patients also like about prehabilitation is that they become self-reliant to be healthier again, as cancer is happening to them. Now, they regain control over and contribute to themselves.”*
Personal support during prehabilitation program	**x**		**x**	**x**	*PT: “With only a brochure it is difficult to motivate the patient… I think they need personal support, for example from a physical therapist, to maximize the result.”* *D: “We see patients at least three times. We also walk around during the exercise training under supervision of the physical therapist… So if patients have questions they can ask them during that training as well.”*
Preoperative multidisciplinary prehabilitation consultation	**x**	**x**	**x**		*PT: “It would be nice to have a preoperative multidisciplinary consultation to evaluate where the patient is now and what he/she still needs. Than you can discuss whether it makes sense to postpone the operation for a week.”*
Set goals and motivate patients to accomplish them			**x**		*PT: “Patients were given assignments to take home with which they could earn points, and the goal was to score as many points as possible each day.”*

*S*, surgeon, *SN*, specialized nurse; *D*, dietician; *PT*, physical therapist

All but one of the obtained barriers and facilitators could be clustered into three categories of the assessment phase of the OMRU framework: the innovation itself, practice environment, and potential adopters. The one code that could not be categorized into the assessment phase was related to the monitor phase of the framework. In Table 4, identified barriers and facilitating factors are classified based on the systematic assessment phase of OMRU.

**Table 4:** Identified barriers and facilitating factors classified based on the systematic assessment phase of OMRU^1^

Category	Barriers	Facilitators
*Innovation: prehabilitation*		
*Relative advantage*	Contradictory and low quality of scientific evidence for (cost-) effectiveness	Evidence regarding effectiveness of prehabilitation is important for program sustainability
	Costs must be financed immediately while yields are not (directly) clear	Both objective as well as patient reported outcomes are important for program evaluation
	Uncertainty which group benefits (most) from prehabilitation	
	Indirect costs for patients (e.g., travel expenses)	
*Compatibility*	Goal and content of prehabilitation program is unclear	Patients are able to improve their self-reliance instead of just waiting
		Application of prehabilitation fits in hospital strategy
*Complexity and reinvention*	Differences in patients’ resilience and training opportunities	Individualized program
*Observability*	Effectiveness difficult to prove due to heterogeneity of patient population	
	Quality of care for colorectal surgery is already high with low complication rates	
*Trialability*		Adjust patient selection during implementation based on (local) results
*Practice environment: hospital*		
*Physical structure*	Combining appointments is difficult due to different work activities	Combining appointments on a single day
	Operating room planning takes precedence over prehabilitation program	Accessible contact between involved healthcare professionals
	Lack of program organization evaluation	Preoperative multidisciplinary prehabilitation consultation
	Patients are unable to visit hospital frequently	Offering an intervention program close to home
		Implementation of digital tools for interaction and reduction of travel distance
*Workload*	Multidisciplinary consultation is time consuming	Evaluation of individual patients only in case of signaled problems or deviation from program
	Counseling patients is time consuming	
*Available resources*	Healthcare system is not adapted, including availability of paramedics in hospital	Coordination of program and program appointments by a specialized nurse
		Guarantee financial support
*Culture and believes*	Time between operation indication and surgery is too short	Delay surgery if necessary
		Introduce prehabilitation early in trajectory
*Potential adopters: health care professionals and patients*		
*Healthcare professionals*	Healthcare professionals are unaware of (importance of) prehabilitation program	Include skeptical healthcare professionals in prehabilitation team from the adoption phase
*Patients*	The idea that sedentary behavior is necessary when cancer is diagnosed	Set goals and motivate patients to accomplish them
	The idea that tumor should be removed as soon as possible	Introduce prehabilitation as part of regular care
		Insight in movement pattern
		Awareness regarding impact of surgery on physical condition
		Incorporate social environment to facilitate patient with prehabilitation program
		Group activities to exchange experiences and motivate peers
		Personal support during prehabilitation program
*Transfer strategies: diffusion, dissemination and implementation of prehabilitation*		
*Local champions*		An ambassador should persuade, enthuse, and unite coworkers

^1^*OMRU*, Ottawa Model of Research Use (OMRU) framework. The framework proposes to study six key components: innovation; environment; adopters; strategies for transferring evidence into practice; the use of evidence; and health-related and other outcomes of the process. These components are connected to each other through the process of evaluation [23]. The framework guides assessment of potential barriers and facilitators to prehabilitation with regard to the innovation (prehabilitation), environment (hospital), adopters (health care professionals and patients), and also the strategies that interviewees identified for the implementation of prehabilitation

### The innovation: prehabilitation

Barriers and facilitators of innovation by prehabilitation were mostly related to the relative (dis)advantages, compatibility, complexity and reinvention, observability (the degree to which the results of prehabilitation are visible to others), and trialability (the degree to which prehabilitation may be experimented with on a limited basis). Contradictory and low-quality scientific evidence for the (cost-)effectiveness of prehabilitation was frequently mentioned. Especially in combination with (high) immediate costs and no directly measurable or visible yields, it was often concluded that advantages of prehabilitation were unclear. Next, heterogeneity of the patient population together with the already high quality of colorectal cancer care surgery and low mortality and complication rates made it difficult to prove effectiveness on a group level. Furthermore, the perceived complexity of prehabilitation and differences in patients’ resilience and training opportunities (i.e., a “one size fits all” prehabilitation program would not work) was seen as barrier.

However, evidence concerning effectiveness of prehabilitation for both objective and patient-reported outcomes could facilitate program sustainability. Insights into effects on individual patient level are also important. Innovation could be further optimized by offering personal programs explicitly. Moreover, the personal experience of added value of prehabilitation was mentioned as an important facilitator, as prehabilitation aligns with patients’ perceived needs to improve self-reliance through prehabilitation rather than passively waiting for surgery.

### Practice environment: the hospital

Barriers and facilitators in the hospital environment where prehabilitation is initiated were mostly related to physical structure, workload, available resources, personalities involved, and culture and beliefs. Identified barriers were mainly logistic. Some patients were not capable to visit the hospital frequently, while combining prehabilitation appointments with different healthcare professionals on a single day was also considered to be difficult because of different work activities of involved healthcare professionals. Also, the combination of counseling patients for prehabilitation and an additional multidisciplinary consultation was seen as time-consuming. The lack of structural program implementation evaluation in a team meeting to identify and resolve experienced problems was mentioned as well. Although the solution of an additional meeting is considered time-consuming, it was thought of as enhancing program sustainability and team building. Furthermore, the timing of surgery was identified as a logistic problem. The inflexible and rapidly changing operation room planning would often take priority over the prehabilitation program, resulting in an early termination of the prehabilitation program. At the same time, national quality indicators [26] state that treatment should take place within 6 weeks of diagnosis, making the time window for prehabilitation often (too) short.

Identified facilitators for the practice environment included combining patient appointments as it would not only lead to a decrease in the number of hospital visits for patients but could also ensure accessible contact between involved healthcare professionals. In addition, offering an intervention program close to home and implementation of digital tools were suggested options to reduce travel distances and facilitate patients’ compliance. Contact through multidisciplinary consultation in order to identify eligible patients and monitor a patients’ progress was identified as facilitator. To partially overcome the problem of time-consuming extra multidisciplinary consultations, it was stated that evaluation of individual patients may only be necessary in case of problems or deviation from the program. The availability of a dedicated nurse specialist who would coordinate the prehabilitation program and various program appointments was deemed important and the guarantee of financial support was seen as an important prerequisite. In order to overcome the timing of surgery, it was stated that it should be possible to delay the procedure if deemed necessary due to patient’s performance status. At last, prehabilitation should be introduced early in the diagnostic trajectory to create sufficient time for prehabilitation while still meeting the national guidelines for timely treatment after diagnosis.

### Potential adopters: health care professionals and patients

Participating healthcare professionals identified themselves as well as patients as early adopters. Barriers and facilitators were related to attitudes, knowledge motivation, skills, and current practices. The unawareness of the importance and possibilities of a prehabilitation program by healthcare professionals was an important barrier. Including skeptic healthcare professionals early in the adoption phase of the innovation could facilitate and overcome this. With regard to patients, the dominating ideas about illness behavior were detrimental as they often believe that sedentary behavior is necessary when cancer is diagnosed. Also, patients believe that the tumor needs to be removed as soon as possible after diagnosis. However, according to the interviewees, patients are often unaware of the impact of surgery on their physical and mental condition and therefore, creating awareness of this impact is a facilitating factor. Patient’s gaining insights in their movement patterns and being able to set personal goals as well as including their social environment could all potentially facilitate adoption by patients. Also, group activities where patients would be able to exchange experiences and motivate peers were identified as a facilitating factor.

### Transfer strategies: diffusion, dissemination, and implementation of prehabilitation

In the monitoring phase, an ambassador, who could persuade, enthuse, and unite coworkers, is necessary for the diffusion, dissemination, and implementation of prehabilitation in the hospital for the long term. This ambassador is preferably a medical specialist.

## Discussion

The aim of this study was to explore barriers and facilitators regarding the implementation of prehabilitation in colorectal cancer surgery as expected and perceived by involved healthcare professionals. Important barriers included the conflicting scientific evidence on (cost-)effectiveness of prehabilitation, the inability of patients to follow a predefined hospital-based prehabilitation program (due to lack of personalized programs or inflexibility of “prescribed” prehabilitation) and the complex logistic organization of the program. Besides, unawareness of (importance of) the prehabilitation program among both healthcare professionals and patients and incorrect ideas of patients about what is important in the preoperative phase were mentioned as serious barriers. Important facilitators were the ability to offer a personalized prehabilitation program for each individual, availability of a program coordinator, and involving skeptical colleagues from the start of the implementation. For transferring prehabilitation within the practice environment, an ambassador was deemed as an important facilitator.

To implement an innovation such as prehabilitation in clinical practice, an individualized program with regard to content, duration, and setting is needed [27]. In order to create more patient-centeredness, questions including what, when, where, who, and why should be taken into account while developing future prehabilitation programs [28]. Additionally, performing a comprehensive geriatric assessment preoperatively can be useful to select patients and increase both adherence and effectiveness of prehabilitation [29].

Furthermore, implementation of prehabilitation requires adjustments in the hospital as practice environment. Local adjustments in the organization of preoperative colorectal cancer care pathways are needed to create availability of dedicated resources and time for involved healthcare professionals. The presence of a program coordinator, for example an oncology nurse, can facilitate effective implementation [30, 31]. This program coordinator can overview the program and signals arising problems on both organizational and patient level. Costs of the additional resources for lifestyle-initiated programs must be guaranteed from the start of implementation, if prehabilitation is indeed (cost-)effective [32]. Financing of these costs should be considered on both hospital and national level [33].

Another adjustment in the organization of preoperative colorectal cancer care should be the possibility to lengthen the time interval between operation indication and surgery, which could serve as a protective time interval to battle negative oncological outcomes [34]. As the mandatory standards [26] and operation room planning currently determine the time between indication and actual surgery, it should rather be the surgeon determining (extended) time until surgery based on the patients’ physical condition and nutritional status and the ability to improve this by prehabilitation.

Because healthcare professionals and patients are not passive recipients of prehabilitation, implementation and adoption of the program should be seen as a transition process rather than an event. In other words, it is important that adopters in the preadoption stage are aware of the innovation. This implies that what prehabilitation does, how to use it, and how it affects the adopter personally should be incorporated [35]. Moreover, skeptics in the surgical pathway need to be included in the prehabilitation team and early in the adoption phase to convince them of the potential merits of prehabilitation and to ensure appropriate information provision towards patients [27, 36].

If potential benefits of prehabilitation remain unclear for recipients, transforming care towards more integration is difficult, and consequently, demonstration of efficacy will fail due to low program adherence. Physicians in particular are the principal players to break this vicious circle by either supporting or opposing successful transformative efforts [37]. Therefore, physicians’ leadership is essential to facilitate diffusion, dissemination, and implementation of prehabilitation both on micro (clinical integration), meso- (professional and organizational integration), and macro- (system integration) levels [38].

## Strengths and limitations

To our knowledge, this is the first qualitative study performed by a multidisciplinary research team with healthcare professionals involved in preoperative colorectal cancer care from different disciplines and hospitals. This provided important insights regarding perceived issues and promotors by implementing prehabilitation from clinical experiences. Although the number of hospitals which have implemented prehabilitation is limited in the Netherlands, many barriers and facilitators for local implementation of prehabilitation in colorectal cancer surgery in research setting were mentioned by multiple healthcare professionals, and thematic saturation was reached as planned. Above, at least theoretical generalizability has been achieved, as all mentioned barriers and facilitators could be placed in the selected framework [39]. As prehabilitation was not part of daily care yet in participating hospitals, implementation regarding perceived barriers and facilitators in daily care instead of a research setting could not be elaborated on.

Previous studies, interviewing patients, highlighted already the importance of appropriate information provision and an accessible personalized prehabilitation program [27, 28, 36, 40]. Nevertheless, the perspective of healthcare professionals on barriers and facilitators at patient level is also of added value [18].

In this study, open coding was independently performed by the first and second author and differences in outcomes were discussed during a group meeting where barriers and facilitators were classified as well. By using direct content analysis, the data collection can become biased by emphasizing this theory [41]. However, the theoretical framework was selected after conducting and coding interviews and therefore overemphasis of the theoretical framework is expected to be minimal. In addition, the use of the OMRU framework guided the discussion of findings, allowing for more explicit recommendations.

## Future research

Although clear and unambiguous evidence of effectiveness is necessary, this will be difficult to obtain for a complex and environment-dependent intervention like prehabilitation, especially if the implementation rate is unsatisfactory. Consequently, individual randomized clinical trials, representing the reference standard, may not be applicable [42]. Instead, pragmatic trials, producing results that can be generalized and applied in routine practice setting, are more appropriate [43]. It would be useful to implement prehabilitation in phases, parallel to monitoring the adoption process and ensuring data-driven continuous improvement [23].

Future trials should perform a preplanned process evaluation including patient experience alongside the effect evaluation to assess fidelity and quality of implementation, clarify causal mechanisms, and identify contextual factors associated with variation in outcomes, resulting in more efficient adaptation, development, and implementation of prehabilitation [44, 45]. A preplanned process evaluation could for example make clear which patient group benefits the most of prehabilitation, especially because prehabilitation programs should be tailor-made and benefits are predominantly patient-specific. Besides, the focus on the process and context of prehabilitation could generate additional hypotheses about mechanisms of success or failure [35]. Furthermore, collaboration between local initiatives and the use of standardized outcome instruments should be emphasized [46].

When evidence regarding effectiveness of prehabilitation is properly displayed, this could persuade skeptics and facilitate the acquisition of financial support, to create a broad-based willingness to implement prehabilitation by both healthcare organizations and healthcare professionals [35]. Future prehabilitation programs should also optimize feasibility, e.g., deliver prehabilitation programs close to home and use digital tools, which were mentioned in this study as facilitators. Finally, the benefits of a longer prehabilitation program, combined with rehabilitation program after surgery, should be further investigated.

In conclusion, important barriers to prehabilitation implementation are mainly related to the intervention being complex, relatively unknown and only minimally evaluated in research settings. The need for clear and unambiguous evidence is however at odds with implementation issues, even in research context, due to negative attitudes of skeptical professionals towards prehabilitation, limited organizational flexibility (e.g., inability to combine appointments), conflicting guidelines (e.g., strict operation timeframe), and patient cognitions (e.g., need for sedentary behavior in illness). Therefore, physicians’ leadership is needed to transform care towards more integration of prehabilitation on micro-, meso-, and macro-levels. The implementation should be phased, with the possibility to adapt the intervention to the variety of real-life contexts and to test its effectiveness in daily practice. Above, the possibility to offer a personalized prehabilitation program will increase willingness to participate in both patients and professionals. By strengthening prehabilitation programs and evidence of their efficacy using these recommendations, it should be possible to enhance both the pre- and postoperative quality of life for future colorectal cancer patients.

## Data Availability

The data and material collected for this article are available on request.

## Code availability

No applicable.
